# Academic anxiety and cognitive reflection in neurodivergence based on evidence from a large international sample

**DOI:** 10.1038/s41598-025-21504-6

**Published:** 2025-10-27

**Authors:** Sheeza Mahak, Stephanie Malone, Mahmoud Elsherif, Christopher J. Hand, Kinga Morsanyi

**Affiliations:** 1 Centre for Mathematical Cognition, Department of Mathematics Education, Loughborough University, Loughborough, UK; 2 Griffith Institute for Educational Research, Griffith University, Brisbane, Australia; 3 School of Psychology, University of Birmingham, Birmingham, UK; 4 School of Psychology and Vision Sciences, University of Leicester, Leicester, UK; 5 School of Education, University of Glasgow, St Andrew’s Building, 11 Eldon Street, Glasgow, G3 6NH UK

**Keywords:** Neurodiversity, Academic anxiety, Mathematics anxiety, Statistics anxiety cognitive reflection, Higher education, Many labs, Psychology, Human behaviour

## Abstract

Anxiety is commonly experienced by neurodivergent individuals participating in higher education, related to both cognitive performance and academic achievement. Whereas certain anxieties in neurodivergence (e.g., social and general anxiety) have received more attention, scant research has considered mathematics and statistics anxiety. In this study, 1383 university students (679 neurotypical, 704 neurodivergent individuals)—matched on age, gender, education level, and country of origin—completed measures assessing various types of academic-related anxiety, attitudes toward mathematics, self-efficacy, and cognitive reflection. Results showed that neurodivergent students exhibited higher anxiety levels across measures, including mathematics and statistics anxiety. However, only cognitive and somatic anxiety, and social anxiety were related to neurodivergent status after taking into account the effects of the other measures. Despite differing anxiety levels (which showed variability between different neurodivergences), neurodivergent and neurotypical students in general were equivalent in cognitive reflection scores, suggesting similar levels of analytic thinking skills, including reasoning about mathematical content. In both the neurotypical and neurodivergent groups, higher levels of cognitive reflection were associated with male gender, less fear of negative evaluation, and lower mathematics and creativity anxiety, replicating and extending previous work. Our findings have implications for supporting neurodivergent individuals in academic settings and other sectors.

**Supplementary Information:**

The online version contains supplementary material available at 10.1038/s41598-025-21504-6.

## Introduction

The ability to analyse, interpret, and be critical about quantitative data is important for daily life and employment. These skills are used when completing tasks such as managing household budgets, and making informed decisions about healthcare, based on available health statistics^1,2^. Many people, however, experience an intense negative state of emotional arousal (i.e., anxiety) associated with the thought, or act, of engaging in mathematics and statistics, with 30.6% of secondary school students reporting feeling nervous when doing mathematics^3^. Moreover, neurodivergent learners appear especially vulnerable, reporting heightened anxiety in academic settings^4^. This fear or worry not only adversely affects academic performance but can also impact career opportunities and choices^5^.

### Academic-related anxiety and neurodivergence

To date, the majority of research studies on academic-related anxieties such as test, mathematics and statistics anxiety have focused on neurotypical individuals (i.e., individuals where functions, behaviours, and processing are characteristic of a typical brain or mind^6^) or samples representing the general population, and it is unknown whether research findings on academic-related anxiety generalise to neurodivergent individuals^i^ (e.g., dyslexic, attention dysregulation hyperactivity development, from here on ADHD^7^, and autistic individuals).

Neurodivergence is an umbrella term, encompassing individuals who have cognitive abilities which differ from neurotypicals in one or more areas, such as language, reading, and movement^8^. Neurodivergent adults tend to face more challenges in mathematical and statistical ability (mathematics difficulties^9^; mathematics problem solving^10^) than neurotypical individuals. Despite this difference in attainment, neurodivergent individuals are encouraged to reach a similar level of understanding of mathematics and statistics as neurotypical individuals, with research providing little insight into how to overcome individual and systemic barriers to mathematics and statistics development^11^.

While research on dyscalculia has predominantly targeted core mathematical deficits^12^, far less attention has been paid to anxiety and performance challenges in other neurodivergent populations (e.g., ASD^13^, ADHD^14^, and dyslexia^15^). Therefore, the current study aims to fill this gap by comparing neurotypical and neurodivergent individuals on a comprehensive set of anxiety measures, including mathematics and statistics anxiety, test anxiety, social anxiety, intolerance of uncertainty, and fear of negative evaluation, as well as attitudes and performance indicators such as self-efficacy and attitude towards mathematics. Extending on this, the study also aims to examine the predictors of cognitive reflection^16^ among both neurotypical and neurodivergent groups.

### Mathematics anxiety in neurodivergent populations

A negative relationship between mathematics/statistics anxiety and mathematics/statistics performance has been identified across countries^17^ and genders^18^, with Barroso et al.^19^ reporting that gender, race and country *do not* moderate the strength of this relationship. In contrast, grade level, mathematics ability level, mathematics topics of anxiety scales, and types of mathematics assessments *do* moderate this relationship^19^. Research has shown that neurodivergent individuals are more likely to experience challenges in mathematics than their neurotypical peers, with this observed for autistic individuals^10^, ADHDers^20^, and dyscalculic people^21^. For dyslexic individuals, a ‘spiky’ profile was observed, where challenges are seen in some, but not all, aspects of mathematics^11^. It may be that these challenges in mathematics contribute to higher levels of anxiety for neurodivergent individuals as a group, compared to their neurotypical peers, which, in turn, impacts mathematical performance^22,23^. This higher level of anxiety for neurodivergent individuals has been supported by some research, including Canu et al.^24^, who observed greater levels of mathematics anxiety for college students with ADHD compared to their non-ADHD peers. Devine et al.^12^ reported that dyscalculic children were twice as likely to experience high levels of mathematics anxiety than their neurotypical peers. Despite these findings, limited research has examined the mathematics- and statistics anxiety levels of neurodivergent people, even though it is well-documented that various forms of anxiety often co-occur with neurodivergences^25,26^. Addressing this issue is crucial, as although a significant proportion of neurotypical people are affected by mathematics and statistics anxiety, neurodivergent students may be even more likely to encounter additional challenges in these areas, for two reasons. First, mathematics learning differences often co-occur with other developmental neurodivergences (e.g., dyslexia^9^). Second, neurodivergent individuals often experience higher levels of anxiety in various situations^12,25–29^.

### Anxiety beyond the context of mathematics and statistics learning

In addition to the specific challenges posed by mathematics and statistics anxiety, neurodivergent learners often experience a range of related anxieties that may impact on their academic performance. For example, they often experience elevated cognitive and somatic anxiety, which correlates with greater severity in both ADHD^27,28^ and autism^31,32^. In autistic individuals, for example, emotional distress is linked to heightened physiological arousal compared to neurotypical peers^31,32^, which can compromise both cognitive flexibility and social functioning^33^. Many neurodivergent learners also suffer from social anxiety and fear of being negatively evaluated, further disrupting their ability to engage confidently in academic settings and assessments^34^. Another common manifestation of anxiety among the neurodiverse population is an intolerance of uncertainty when faced with unpredictable or ambiguous situations. Individuals may react with frustration or task avoidance, and may be unable to cope with the associated distress (ASD^35^ and ADHD^36^). Cognitive models suggest that elevated anxiety levels have a negative impact on academic outcomes by depleting attentional resources and reducing mental flexibility^27,28,37^. Indeed, a systematic review found that autistic participants show notably reduced cognitive flexibility, a capacity closely tied to creative problem solving^29^. Students with learning difficulties have also reported higher levels of test anxiety compared to neurotypical groups^38^.

Other factors closely linked to both mathematics performance and mathematics/statistics anxiety are self-efficacy and attitude towards mathematics. Self-efficacy is defined as the belief in one’s ability to achieve a desired outcome^39^, and it has been shown to be negatively associated with mathematics anxiety^40^, whilst being positively associated with performance^41^. In turn, negative attitudes towards mathematics have been consistently linked to lower performance^42^. Research involving neurodiverse populations has reported similar patterns, and also indicated that neurodiverse individuals may be vulnerable to exhibiting lower self-efficacy^43^ and showing a negative attitude towards mathematics^15,44^, which, in turn, can have detrimental consequences for academic achievement^43^. Despite the clear impact of these intertwined anxieties and self-efficacy on learning, research examining their presence in neurodivergent groups, including autism, ADHD, dyslexia, dyscalculia, and dyspraxia, remains scarce.

To further understand the presentation of these varying forms of anxiety for neurodivergent individuals, the current study focused on identifying differences in mathematics and statistics anxiety, together with self-efficacy, attitude towards mathematics, performance anxiety, social and cognition-related anxiety, and other academically-related anxiety levels among neurodivergent individuals from a large and diverse sample.

### Cognitive reflection and neurodivergence

The Cognitive Reflection Test (CRT)^16^ involves responding to word-based problems with a numerical content, and measures a person’s ability to resist intuitive response tendencies in favour of a more effortful, deliberative response, which has previously been identified as being correlated with academic performance for neurotypical individuals^45^. While the CRT includes mathematical word problems, it is frequently used to measure decision-making skills and rational thinking^16,46^. Nevertheless, performance on the CRT is associated with numerical skills^47^, as well as mathematics anxiety^48,48,50^, even after controlling for the effects of test anxiety^50^, evidencing a strong mathematical element. A notable feature of the CRT is that although the problems are open-ended, they consistently elicit two primary types of responses: the correct, deliberative answer and a typical incorrect answer that stems from an intuitively compelling, but misleading initial impulse. Other types of incorrect responses are rare. This consistent elicitation of intuitive incorrect responses makes the CRT a powerful tool for assessing individual differences in participants’ ability to override incorrect intuitions (and to display cognitive reflection). For this reason, the CRT can be used to investigate whether neurodivergent individuals share the same incorrect mathematical intuitions as neurotypical people.

To the best of our knowledge, performance on the CRT has only been investigated in the case of autistic individuals, and these studies have resulted in mixed findings. Brosnan et al.^51^ and Brosnan and Ashwin^52^ reported that autistic participants gave fewer intuitive responses to the CRT and produced a higher proportion of correct, deliberative responses. However, these findings were not replicated by Taylor et al.^53^ and Morsanyi and Hamilton^54^, who reported no difference between autistic and neurotypical participants in either intuitive or deliberative responding on the CRT. In terms of performance on the CRT among neurodivergent individuals in general, given the connection between cognitive reflection and both mathematics skills^15^ and mathematics anxiety^55^, it could be expected that neurodivergent individuals might show reduced performance, relative to neurotypical participants.

### The current study

Measures of academic-related anxiety have been investigated only recently in a large sample, and have been validated cross-culturally^56^. As a result of scaling up data collection, multiple laboratories can work together to produce larger sample sizes than is achieved with a small-scale science approach by individual laboratories^57^. This allows for greater statistical power, which is necessary to investigate small effects and interactions, and to conduct analyses where multiple comparisons are required (e.g., Vaidis et al.^57^). In addition, the construct and measures of mathematics and statistics anxiety have been investigated primarily in Western, Educated, Industrious, Rich and Democratic (WEIRD) populations. By contrast, a big-team science approach allows us to recruit more diverse and representative populations from the Global North and the Global South^8^. Indeed, statistics is a requirement not only in the Global North but around the globe, as it is necessary to navigate a data-driven world^59^. However, available findings are limited, as the majority of studies have focused either on neurotypical participants, neurodivergent participants mixed with neurotypical participants, or neurodivergent participants in the Global North with small samples. Thus, available data, including neurodivergent participants, is sparse. It is therefore important to scale up and diversify data collection to include neurodivergent participants across the globe.

We adopted a data-driven approach to identify and understand the relation between academic-related anxiety and achievement in these areas for neurodivergent individuals, using data from the Statistics and Mathematics Anxieties and Related Variables in University Students (SMARVUS^56^) dataset. From this dataset, we included all neurodivergent participants, as well as a neurotypical comparison group, carefully matched to the neurodivergent group on age, gender, education level and country of origin. Participants were assessed on mathematics anxiety, statistics anxiety, test anxiety, social anxiety, cognitive and somatic anxiety, creativity anxiety, intolerance of uncertainty, and fear of negative evaluation, attitudes toward mathematics, general self-efficacy, and analytical thinking (CRT).

The first aim of our study was to compare neurotypical and neurodivergent individuals’ scores on this set of measures. To do so, beyond simple group comparisons, we ran multiple regression analyses to investigate whether these measures could be used to discriminate between neurotypical and neurodivergent students. The second aim of the study was to examine the predictors of cognitive reflection in the case of both neurotypical and neurodivergent individuals. Specifically, we were interested in the relations between cognitive reflection and mathematics anxiety, when the effects of some demographic factors, as well as other forms of anxiety were taken into account. For this, separate hierarchical linear regression analyses were used for deliberative and intuitive responses. We were also interested in the level of deliberate and intuitive responding in neurodivergent samples as compared to the non-neurodivergent population. This question has been investigated in a limited number of studies already, but results have been contradictory. As different types of neurodivergence are associated with different cognitive profiles, we also investigated these questions in specific neurodivergent groups separately.

## Results

Descriptive statistics and independent *t* test results comparing the matched neurotypical and neurodivergent groups on all measures are presented in Table 1.

**Table 1:** Independent t tests comparing the Neurotypical (N = 679) and Neurodivergent (N = 704) groups on all study variables.

	Neurotypical		Neurodivergent			
Measures	M (SD)	Range	M (SD)	Range	p	Cohen’s d
Statistics anxiety	64.05 (19.91)	23–115	68.76 (21.32)	23–115	< 0.001**	0.23
Mathematics anxiety	69.71 (21.01)	24–119	75.14 (22.35)	24–120	< 0.001**	0.25
Cognitive and somatic anxiety	43.24 (13.20)	21–84	48.47 (14.69)	21–84	< 0.001**	0.37
Test anxiety	57.21 (16.60)	25–100	62.66 (17.09)	25–100	< 0.001**	0.32
Fear of negative evaluation	24.46 (9.23)	8–40	25.85 (9.11)	8–40	0.005**	0.15
Social anxiety	55.39 (15.07)	24–94	57.28 (15.14)	24–96	0.020*	0.13
Creativity anxiety	40.33 (12.52)	16–78	42.29 (12.02)	16–80	0.003**	0.16
Intolerance of uncertainty	33.54 (10.46)	12–60	35.58 (10.38)	12–60	< 0.001**	0.20
General self-efficacy	29.31 (5.46)	9–40	28.35 (5.90)	8–40	0.002**	0.17
Attitude towards mathematics	24.90 (4.70)	7–35	24.37 (4.81)	10–35	0.036*	0.11
CRT (deliberative response)	1.05 (1.10)	0–3	1.00 (1.12)	0–3	0.362	0.05
CRT (intuitive response)	1.70 (1.12)	0–3	1.70 (1.13)	0–3	0.972	< 0.01

*p < 0.05, **p < 0.001; CRT = Cognitive Reflection Test.

There was variability of scores on all measures, and the groups significantly differed on all measures of anxiety, as well as on self-efficacy and attitudes towards mathematics, suggesting a neurotypical advantage on all of these measures. The effect sizes of these group differences were small to moderate. However, there was no difference between the groups on the CRT, including both deliberative and intuitive responses.

Subgroup analyses for ADHD, autism, dyscalculia, dyslexia and dyspraxia are reported in the Supplementary materials (see Tables S5, S8, S11, S14 and S16; OSF pagehttps://osf.io/ypa8z/), and are summarised in Table 2. These analyses showed variability in the results according to types of neurodivergence. To summarise, students with dyslexia reported higher cognitive and somatic anxiety than matched controls. Interestingly, dyslexic students also gave significantly more deliberative and fewer intuitive responses to the CRT than matched controls. Students with ADHD also displayed higher cognitive and somatic anxiety than controls, as well as higher test anxiety and lower self-efficacy. Dyscalculic students showed elevated statistics and mathematics anxiety, as well as more fear of negative evaluation and less positive attitudes towards mathematics. They also gave fewer deliberative responses to the CRT than controls. The number of autistic and dyspraxic students was low in our sample. Thus, only those group differences that were associated with large effect sizes reached significance. Autistic students showed higher levels of intolerance of anxiety than matched controls, whereas other group differences were non-significant. Dyspraxic students also displayed higher levels of intolerance of uncertainty, as well as higher test anxiety than controls.

**Table 2:** Cohen’s d effect sizes for comparisons between neurodivergent students and matched neurotypical controls on our measures of interest (significant differences are marked by asterisks and highlighted in bold; positive values indicate a higher score in the neurodivergent sample).

	Dyslexia *(n* = *181)*	ADHD *(n* = *293)*	Dyscalculia *(n* = *57)*	Autism *(n* = *16)*	Dyspraxia *(n* = *14)*
Statistics anxiety	< 0.01	− 0.08	0.38*	0.28	0.41
Mathematics anxiety	− 0.04	0.02	0.77**	0.61	0.29
Cognitive and somatic anxiety	0.22*	0.34**	0.21	0.24	0.72
Test anxiety	− 0.01	0.24*	0.20	0.38	1.37**
Fear of negative evaluation	− 0.09	0.12	0.43*	0.60	0.71
Social anxiety	− 0.10	0.02	0.36	0.54	0.29
Creativity anxiety	− 0.03	0.02	0.17	0.36	0.14
Intolerance of uncertainty	0.03	0.05	0.31	1.09**	0.84*
General self-efficacy	− 0.19	− 0.19*	− 0.06	− 0.45	− 0.10
Attitude towards mathematics	− 0.11	− 0.04	− 0.44*	0.29	0.22
CRT (deliberative response)	0.37**	0.13	− 0.51**	− 0.40	0.17
CRT (intuitive response)	− 0.33**	− 0.14	0.13	0.39	− 0.23

*p < 0.05, **p < 0.01; CRT = Cognitive Reflection Test.

### Relationships between anxiety measures, self-efficacy, attitudes towards mathematics and cognitive reflection

Our next analysis aimed to investigate the relationships between various types of anxiety, self-efficacy, mathematics-related attitudes, and cognitive reflection, while controlling for the effects of age and gender. These analyses were run separately for the neurodivergent and neurotypical groups. The partial correlations controlling for age and gender are presented in Table 3. (N.B., we also ran bivariate correlation analyses without the control variables, but this did not substantially impact the results in either group).

**Table 3:** Partial correlations (controlling for age and gender) between all measures for the Neurotypical (upper triangle) and Neurodivergent groups (lower triangle).

	1	2	3	4	5	6	7	8	9	10	11	12
**1.** Statistics anxiety	–	0.75***	0.43***	0.54***	0.42***	0.49***	0.50***	0.44***	− 0.28***	− 0.28***	− 0.19***	0.17***
**2.** Mathematics anxiety	0.76***	–	0.40***	0.54***	0.39***	0.40***	0.45***	0.35***	− 0.26***	− 0.29***	− 0.29***	0.24***
**3.** Cognitive and somatic anxiety	0.48***	0.43***	–	0.63***	0.55***	0.61***	0.46***	0.59***	− 0.37***	− 0.26***	− 0.07	0.07
**4.** Test anxiety	0.60***	0.57***	0.67***	–	0.50***	0.55***	0.48***	0.54***	− 0.30***	− 0.28***	− 0.15***	0.13***
**5.** Fear of negative evaluation	0.43***	0.33***	0.60***	0.51***	–	0.58***	0.32***	0.49***	− 0.31***	− 0.14***	0.07	− 0.07
**6.** Social anxiety	0.53***	0.44***	0.60***	0.53***	0.60***	–	0.52***	0.52***	− 0.37***	− 0.23***	− 0.06	0.06
**7.** Creativity anxiety	0.55***	0.52***	0.47***	0.49***	0.38***	0.49***	–	0.48***	− 0.27***	− 0.31**	− 0.21***	0.16***
**8.** Intolerance of uncertainty	0.40***	0.34***	0.53***	0.49***	0.54***	0.54***	0.44***	–	− 0.24***	− 0.22***	− 0.10**	0.10**
**9.** General self-efficacy	− 0.22***	− 0.17***	− 0.31***	− 0.26***	− 0.28***	− 0.28***	− 0.18***	− 0.22***	–	0.32***	− 0.06	0.05
**10.** Attitude towards mathematics	− 0.36***	− 0.32***	− 0.26***	− 0.29***	− 0.15***	− 0.21***	− 0.40***	− 0.22***	0.23***	–	0.15***	− 0.11**
**11.** CRT (deliberative responses)	− 0.26***	− 0.29***	− 0.12**	− 0.20***	0.02	− 0.09*	− 0.24***	− 0.07	0.02	0.17***	–	− 0.89***
**12.** CRT (intuitive response)	0.23***	0.28***	0.09**	0.17***	− 0.02	0.07	0.18***	0.02	0.03	− 0.10**	− 0.85***	–

Correlations were significant at *p < 0.05, **p < 0.01, ***p < 0.001 (2-tailed). CRT = Cognitive Reflection Test.

Overall, we found very similar trends for the neurodivergent and neurotypical groups. The different types of anxiety measures showed significant, moderate-to-strong positive associations. Additionally, higher anxiety levels were generally associated with lower self-efficacy, more negative attitudes towards mathematics, and poorer performance on the CRT, although no significant relation was found between cognitive reflection and fear of negative evaluation and self-efficacy in either group.

### Regression analyses predicting group membership

To understand which measures were the most predictive of group membership (i.e., neurotypical vs. neurodivergent), we ran a hierarchical regression analysis. The scores for all measures were scaled and centred before the analysis.

In the regression model, we used neurotypical/neurodivergent as the outcome variable (0 = neurotypical; 1 = neurodivergent), with all other measures as predictors, and added age (as continuous variable) and gender (− 1 = male; 1 = female) as covariates. Given that certain variables (especially the anxiety measures) were highly correlated, we also computed multicollinearity statistics. The analysis showed that the VIF value for each predictor was acceptable (VIF < 2.98). Additionally, we checked if all models met the conditions of independence of residuals. The tests indicated a violation of residuals of normal distribution and homoscedasticity. However, given the large sample sizes, these violations were not considered consequential^60^.

A significant effect was observed in the model (*F* (13, 1359) = 5.49, *p* < 0.001, *η*^2^ = 0.05). The analysis showed that, after controlling for the non-significant effects of age and gender, and all other types of anxiety and attitudes, only higher cognitive and somatic anxiety and social anxiety were independently associated with a higher probability of being in the neurodivergent group (see Table 4). We also ran the analysis without centring and scaling measures, and this did not have a substantial impact on the results.

**Table 4:** Hierarchical regression predicting group membership by demographics, anxiety measures and CRT.

	Dependent variable: Neurodivergent status (0, 1)				
	*b*	*beta*	*t*	*p*	*Adj R*^*2*^
Step 1					0.001
Age	0.01	0.02	0.68	0.497	
Gender	− 0.01	− 0.01	− 0.42	0.672	
Step 2					0.041
Age	0.03	0.03	1.23	0.219	
Gender	− 0.03	− 0.04	− 1.52	0.128	
Statistics anxiety	< 0.01	0.02	0.46	0.646	
Mathematics anxiety	0.01	0.06	1.28	0.201	
Cognitive and somatic anxiety	< 0.01	0.19	4.68	< 0.001**	
Test anxiety	< 0.01	0.08	1.87	0.062	
Fear of negative evaluation	< 0.01	− 0.05	− 1.42	0.157	
Social anxiety	< 0.01	− 0.09	− 2.29	0.022*	
Creativity anxiety	< 0.01	− 0.02	− 0.67	0.502	
Intolerance of uncertainty	< 0.01	0.01	0.18	0.856	
General self-efficacy	< 0.01	− 0.04	− 1.22	0.223	
Attitude towards mathematics	< 0.01	0.01	0.32	0.746	
Cognitive reflection test	0.01	0.02	0.52	0.604	

*p < 0.05, **p < 0.001; *b* = unstandardized regression weights, *beta* = standardized regression weights.

We also performed subgroup analyses for those groups where sample sizes were over 100 for the combined neurotypical and neurodivergent samples (i.e., for dyslexia, ADHD and dyscalculia). These are reported in the Supplementary Materials (Tables S6, S9, S12). In the case of the dyslexic group, both cognitive and somatic anxiety and analytic performance on the CRT emerged as significant, positive predictors of group membership (*Adj R*^*2*^ = 0.06*).* In the case of ADHD, cognitive and somatic anxiety, as well as test anxiety were significant positive predictors of group membership, whereas social and statistics anxiety were negative predictors (*Adj R*^*2*^* = *0.05*).* In the case of the dyscalculia, only mathematics anxiety was a significant predictor (*Adj R*^*2*^ = 0.11).

### Regression analyses predicting CRT performance

Although we found no differences between groups in cognitive reflection, we also conducted a hierarchical regression analysis separately for each group (i.e., neurotypical and neurodivergent) to investigate the predictors of cognitive reflection (Table 4). This regression enabled us to determine whether anxiety and other factors had a similar relationship with CRT performance in neurodivergent individuals, as compared to neurotypical individuals. Consistent with the previous method, all scores were scaled and centred prior to analysis. Age (a continuous measure) and gender (coded as − 1 = male, 1 = female) were included in Block 1, while all other measures were entered in Block 2. For analyses predicting group membership for intuitive response (See supplementary material, Table S3). Additionally, group-level analyses were conducted to predict group membership for cognitive reflection in other neurodivergent groups, including ADHD (see Supplementary Material, Table S8), dyscalculia (see Supplementary Material, Table S12), and dyslexia (see Supplementary Material, Table S6) (Table 5).

**Table 5:** Hierarchical regression predicting CRT deliberative response performance in each group.

	Neurotypical group						Neurodivergent group			
	*b*	*beta*	*t*	*p*	*Adj R*^*2*^	*b*	*beta*	*t*	*p*	*Adj R*^*2*^
Step 1					0.03*					0.05*
Age	0.02	0.01	0.29	0.768		0.05	0.03	0.85	0.398	
Gender	− 0.25	− 0.19	− 4.90	< 0.001**		− 0.31	− 0.23	− 6.13	< 0.001**	
Step 2					0.18*					0.17*
Age	0.05	0.03	0.88	0.377		0.03	0.02	0.54	0.587	
Gender	− 0.21	− 0.16	− 4.26	< 0.001**		− 0.23	− 0.17	− 4.62	< 0.001**	
Statistics anxiety	0.00	0.07	1.19	0.233		− 0.01	− 0.10	− 1.64	0.100	
Mathematics anxiety	− 0.02	− 0.35	− 6.25	< 0.001**		− 0.01	− 0.16	− 2.73	0.007**	
Test anxiety	0.00	− 0.04	− 0.68	0.498		− 0.01	− 0.09	− 1.63	0.103	
Cognitive and somatic anxiety	0.00	− 0.02	0.31	0.754		0.00	− 0.01	− 0.26	0.792	
Fear of negative evaluation	0.03	0.25	5.16	< 0.001**		0.01	0.19	3.83	< 0.001**	
Social anxiety	0.00	− 0.02	− 0.35	0.724		0.00	0.03	0.57	0.572	
Creativity anxiety	− 0.01	− 0.12	− 2.70	0.007**		− 0.01	− 0.14	-2.87	0.004**	
Intolerance of uncertainty	0.00	-0.05	-0.94	0.346		0.00	0.02	0.36	0.723	
General self- efficacy	− 0.03	− 0.15	− 3.81	< 0.001**		− 0.01	− 0.03	− 0.77	0.442	
Attitude towards mathematics	0.02	0.08	2.06	0.040**		0.01	0.05	1.14	0.253	

*p < 0.05, **p < 0.001; *b* = unstandardized regression weights, *beta* = standardized regression weights; CRT = Cognitive Reflection Test.

The results of the regression analyses were similar for both groups, showing that, after taking into account the effect of age and the remaining variables, gender, mathematics anxiety, fear of negative evaluation and creativity anxiety emerged as significant predictors of performance on the cognitive reflection test. In the neurotypical group only, general self-efficacy and attitude toward mathematics were also significant predictors. Interestingly, fear of negative evaluation was positively related to performance on the CRT, whereas self-efficacy was a negative predictor (although these relationships only emerged after controlling for the effect of the other variables, and were not present in the simple correlation analyses). For the sake of completeness, we also report the same analyses for intuitive responses in the Supplementary Materials (Table S3). The results were very similar; gender, mathematics anxiety, and fear of negative evaluation predicted intuitive responding in the CRT for both groups. In the neurotypical group only, self-efficacy was also a significant predictor.

### The moderating effect of neurodivergent status on predictors of performance on the CRT

The previous analyses suggested that although there was much overlap in the predictors of performance on the CRT, there was also some indication of potential differences between neurotypical and neurodivergent groups in the predictors of CRT performance. To statistically assess the presence of any group differences, we conducted an additional hierarchical regression analysis for the combined neurodivergent and neurotypical sample, incorporating interaction terms for neurodivergent status. This enabled us to statistically test whether measures exhibited different associations with CRT performance across the two groups (Table 6). Consistent with the previous method, all scores were scaled and centred prior to analysis. Age (a continuous measure) and gender (coded as -1 = male, 1 = female) were included in Block 1. In Block 2, all other measures were entered (main effects), whereas in Block 3, interaction terms between each predictor and neurodivergent (ND) status were added. We also conducted a similar analysis for intuitive responses (see Supplementary Material, Table S4).

**Table 6:** Hierarchical regression predicting CRT performance; main effects and interactions with neurodivergent status.

	Step 1				Step 2				Step 3			
	*b*	*beta*	*t*	*p*	*b*	*beta*	*t*	*p*	*b*	*beta*	*t*	*p*
Age	0.04	0.02	0.81	0.420	0.04	0.02	0.82	0.410	0.04	0.03	0.98	0.328
Gender	− 0.57	− 0.21	− 7.81	< 0.001**	− 0.44	− 0.16	− 6.29	< 0.001**	− 0.44	− 0.16	− 6.29	< 0.001**
Statistics anxiety					0.00	− 0.02	− 0.48	0.628	0.00	0.07	1.22	0.223
Mathematics anxiety					− 0.01	− 0.25	− 6.12	< 0.001**	− 0.02	− 0.36	− 6.08	< 0.001**
Test anxiety					0.00	− 0.07	− 1.75	0.081	0.00	− 0.03	− 0.62	0.536
Creativity anxiety					− 0.01	− 0.13	− 3.89	< 0.001**	− 0.01	− 0.12	− 2.58	0.010**
Fear of negative evaluation					0.03	0.21	6.24	< 0.001**	0.03	0.25	5.07	< 0.001**
Social anxiety					0.00	0.01	0.36	0.716	0.00	− 0.01	− 0.19	0.846
Cognitive and somatic anxiety					0.00	− 0.02	− 0.45	0.653	0.00	− 0.01	− 0.19	0.846
Intolerance of uncertainty					0.00	− 0.01	− 0.33	0.739	0.00	− 0.04	− 0.90	0.369
General self− efficacy					− 0.02	− 0.08	− 3.05	0.002**	− 0.03	− 0.14	− 3.64	< 0.001**
Attitude towards mathematics					0.01	0.06	2.27	0.023**	0.02	0.10	2.71	0.007**
Statistics anxiety*ND									− 0.01	− 0.32	− 2.09	0.037*
Mathematics anxiety*ND									0.01	0.37	2.46	0.014*
Test anxiety*ND									0.00	− 0.12	− 0.78	0.435
Creativity anxiety *ND									0.00	− 0.06	− 0.48	0.633
Fear of negative evaluation*ND									− 0.01	− 0.08	− 0.77	0.441
Social anxiety*ND									0.00	0.07	0.50	0.618
Cognitive and somatic anxiety *ND									0.00	− 0.01	− 0.08	0.934
Intolerance of uncertainty*ND									0.01	0.10	0.84	0.400
General self− efficacy*ND									0.02	0.25	2.09	0.037*
Attitude towards mathematics*ND									− 0.02	− 0.19	− 1.62	0.106
***Adj R***^***2***^	0.043**				0.171**				0.174**			

*p < 0.05, **p < 0.001; *b* = unstandardized regression weights, *beta* = standardized regression weights; CRT = Cognitive Reflection Test; ND = Neurodivergent status.

The results indicated that mathematics anxiety and self-efficacy were significantly more strongly associated with CRT performance in the neurotypical than in the neurodivergent group. Nevertheless, mathematics anxiety was a significant predictor of CRT performance in both groups, as indicated by the separate regression analyses, whereas self-efficacy only had a significant effect in the case of the neurotypical group. An interaction involving statistics anxiety was also observed; however, this interaction was not associated with significant main effects in either group, and is therefore of limited interpretive value.

## Discussion

This study had two aims: the first was to investigate mathematics and statistics anxiety levels, together with a range of other academic-related anxiety measures, attitudes towards mathematics, self-efficacy, and cognitive reflection, and their inter-relations in a large international sample of neurodivergent and neurotypical university students. The second aim was to analyse the predictors of cognitive reflection among both populations. The two groups were closely matched on age, gender, country of origin and level of education. We also run these analyses separately for the autistic, ADHD, dyslexic, dyscalculic, and dyspraxic groups, when sample sizes were large enough to do so (see Supplementary Materials, Tables S5–S17).

### Anxiety, mathematics-related attitudes and self-efficacy in neurodivergent students

Whereas internalising problems are commonly reported in neurodivergent conditions, specific types of anxiety (beyond general anxiety) are rarely investigated, and sample sizes in these studies tend to be small. Thus, our study offered novel contributions by investigating various types of anxiety in larger and more diverse neurodivergent populations.

As expected, we found that neurodivergent adults (when all diagnostic categories were considered together) displayed higher levels of anxiety (including all types of anxiety measured in our study) and lower levels of self-efficacy, although the effect sizes of these group differences were modest. Despite the presence of group differences in almost all measures, when included in a single regression model, only cognitive and somatic anxiety, and social anxiety discriminated between the neurodivergent and neurotypical groups beyond the effect of the other variables. This indicates that, in general, mathematics- and statistics anxiety and attitudes towards mathematics were not particularly prominent areas of concern relative to other types of anxiety, despite the significant group differences.

Nevertheless, subgroup analyses (see Supplementary Materials, Tables S5–S17) revealed a more nuanced picture. Students with dyslexia and students with ADHD showed higher levels of cognitive and somatic anxiety than neurotypical controls (see also Giovagnoli et al.^61^, and Okyar et al.^62^), whereas group differences in cognitive and somatic anxiety were not found in the case of dyscalculia, autism or dyspraxia. Students with ADHD also displayed higher levels of test anxiety (see also Dan & Raz^63^), and lower self-efficacy. As it could be expected, and in line with the results of previous studies^23^, dyscalculic students showed higher levels of mathematics anxiety than a matched group of neurotypical students. Moreover, mathematics anxiety continued to be a significant predictor of dyscalculic status after controlling for the effects of age and the other study variables, including the effects of cognitive reflection. Dyscalculic students also showed significantly higher levels of statistics anxiety, fear of negative evaluation, and less positive attitudes toward mathematics. Although our autistic sample was small, a significant difference emerged in intolerance of uncertainty relative to the neurotypical group, with a large effect size. A tendency to find it difficult to cope with unexpected or unknown situations can be considered a core characteristic of autism, and elevated levels of intolerance of uncertainty in autism are documented by a large body of literature (see Jenkinson et al.^64^ for a systematic review). Dyspraxic students also displayed higher levels of intolerance of uncertainty, as well as higher test anxiety than controls. Other effects failed to reach significance in this group.

### Cognitive reflection and neurodivergence

Interestingly, no difference between the overall neurodivergent and neurotypical groups was found in cognitive reflection, a measure with a strong mathematics component^39^, which is commonly used as a measure of analytic thinking and decision-making skills. It is interesting to contrast this lack of group difference in analytical thinking with the finding that neurodivergent students displayed higher levels of anxiety (including mathematics and statistics anxiety) and lower levels of self-efficacy.

Despite the lack of difference in cognitive reflection in the overall neurotypical and neurodivergent groups, there was a significant difference between dyscalculic and neurotypical students on the CRT. Specifically, dyscalculic students showed reduced levels of deliberative responses. However, there was no difference in producing intuitive responses between the two groups, suggesting that dyscalculic students shared the same incorrect intuitions related to mathematics as neurotypical students. The subgroup analyses also revealed an unexpected result, with dyslexic students performing significantly better on the CRT than the matched neurotypical control group. However, this result should be interpreted with caution, as performance in the dyslexic group was very close to the population mean, with the matched control group showing lower performance. Another finding that is worth mentioning in relation to performance on the CRT is the lack of difference in performance between the autistic and matched neurotypical group in both deliberative and intuitive responses. This result is in line with Morsanyi and Hamilton^54^ and Taylor et al.^53^, but contrasts with Brosnan et al.^65^ and Brosnan and Ashwin^52^ who reported better performance in autistic than neurotypical participants. This divergence might result from smaller sample sizes, and lack of control between groups relating to demographic and cognitive measures in Brosnan and colleagues’ studies.

In terms of the predictors of performance on the CRT, we replicated previous findings^48,50^ that showed that both gender and mathematics anxiety were important predictors of performance on the CRT, beyond the effect of other forms of anxiety. Nevertheless, the current results extend these earlier findings by considering not just various types of anxiety, but also mathematics-related attitudes, self-efficacy and fear of negative evaluation, in a larger sample, and also replicating earlier results in a neurodivergent group. Indeed, the predictors of CRT performance were very similar in both groups, with the exception that mathematics and statistics anxiety, as well as self-efficacy were significantly more strongly associated with CRT performance in the neurotypical group. Although we can only speculate about the reasons for these differences, it may be the case that in neurodivergent students, mathematics/statistics-related anxiety and self-efficacy are influenced by some factors (for example, communication skills) that are not directly related to mathematics performance. This may explain the weaker relation between these constructs and performance on the CRT. Nevertheless, it should be noted that in the regression analysis, we have already controlled for several potentially relevant constructs, such as social anxiety and fear of negative evaluation.

An additional novel contribution of the current study is to show a relationship between performance on the CRT and creativity anxiety^66^, which was present in both groups, and remained significant after controlling for the effect of all other predictor variables. Although the relation between creativity anxiety (e.g., “Having to solve a problem for which the solution is open-ended”; “Working in a situation where there is an established correct and incorrect way of doing things”) and cognitive reflection may be unsurprising given the format and content of the CRT, this finding enriches our understanding of what the CRT measures, and also contributes to the sparse literature regarding the link between reflection and creativity^67^.

### Applications

Based on the findings presented in this study, it is imperative for educational institutions to recognize and address the unique challenges faced by neurodivergent students^8^. Cognitive and somatic anxiety (i.e., trait anxiety) showed the largest difference between the neurotypical and neurodivergent groups. However, all other types of anxiety were also more common in the neurodivergent group. This included anxiety related to specific subjects, such as mathematics and statistics, as well as test anxiety, creativity anxiety, social and performance anxiety, and fear of negative evaluation. All of these types of anxiety are relevant to studying at university, and could impede on the performance of students. While neurodivergent students exhibited heightened anxiety, overall, they did not differ from neurotypical students in their analytic thinking (i.e., cognitive reflection). Therefore, it is crucial to avoid making assumptions about these students’ capabilities based solely on their anxiety levels. Instructors should adopt inclusive teaching practices that accommodate the diverse needs of neurodivergent students^8,12,15,24–26,30^. This may involve providing flexible assessment options, offering additional support and resources, and creating a supportive classroom environment that minimises anxiety and promotes a positive learning experience. As a result, educators can develop effective strategies to foster the success and wellbeing of neurodivergent students in these critical academic areas.

### Limitations and future research

Although our study had several strengths, including large sample sizes and a geographically and culturally diverse sample, it also had some limitations. Given the cross-sectional design, the present study can only establish relationships between variables, without necessarily implying causality. The terms “predictor” and “outcome” should be interpreted within the models’ context, as bidirectional relationships or reverse causation may be present and warrant further investigation.

Furthermore, because we employed a statistical approach to match neurotypical and neurodiverse samples on key demographic variables, the data we ultimately obtained predominantly represented Western countries (i.e., the UK, Canada, and USA), where there is a higher chance that neurodivergent people have access to diagnosis and support, and entering higher education. Furthermore, as our focus was on anxiety in academic settings in young adults, we focused on highly educated samples. Thus, overall, the composition of our sample restricts the generalisability of our findings to non-WEIRD populations. Whereas the large sample size and cross-cultural data collection is a strength of our approach, future research with more equitably balanced and diverse samples would be necessary to establish the generalisability of our findings to the global neurodivergent population. Focusing on highly educated, high-ability participants, might have also resulted in an underestimation of typical group differences.

Additionally, as our results clearly showed, results combined across different neurodivergent conditions do not necessarily apply to specific groups of neurodivergent individuals. In terms of our neurodivergent-specific analyses, although some of our neurodivergent groups were large (i.e., for ADHD, dyslexia, dyslexia), some other groups (i.e., autism, dyspraxia, and participants with multiple neurodivergent conditions) were too small to run more detailed analyses.

Diagnoses were also based on self-report and were not independently confirmed. Importantly, however, participants received no incentives for self-identifying as neurodivergent, and there was no indication that this would be a focal aspect of the study, which minimised any motivation to over- or under-report diagnostic status. As reporting conferred no advantage, this enhances the credibility of self-reported diagnoses. While we acknowledge the inherent limitations of any self-report data for diagnosis, particularly in research settings, dismissing it entirely overlooks a significant, valid, and reliable source of information, especially from a community-centric perspective.

The neurodivergent sample’s heterogeneity also underscores the importance of considering individual variations in experiences and challenges. Thus, future research should delve into the underlying mechanisms of differences in various forms of anxiety within specific neurodivergences. Disentangling neurodivergence groups and examining the distinctions between different forms of anxiety within these individual groups will lead to more nuanced results and conclusions.

### Conclusion

Our study is the largest and most diverse investigation of mathematics and statistics anxiety, as well as various other types of anxiety and attitudinal measures in neurodivergent conditions to date. It reveals that across a variety of anxiety types (including mathematics and statistics anxiety), neurodivergent individuals experience higher levels of anxiety, although the specific profiles differ across different neurodivergent conditions. This can influence these students’ experiences and performance in a variety of educational and non-educational contexts, which highlights the need for tailored support. Nevertheless, despite a common experience of anxiety, neurodivergent students in general exhibited analytical thinking and problem-solving skills, which were very similar to their neurotypical peers. Our findings relating to specific cognitive and emotional profiles in neurodivergent students can be useful for developing appropriate coping strategies and mechanisms of targeted support. Future research should extend these investigations by further examining condition-specific cognitive and emotional profiles, and offering targeted interventions and support.

## Method

### Openness and transparency

All data and the R code to reproduce the current findings are provided via Open Science Framework^68^. Ethical approval for the project has been obtained from the University of Sussex Sciences and Technology Research Ethics Committee (approval number: ER/JLT26/7). Additionally, all other participating institutions have obtained ethics approval from their local institutional ethics boards before starting data collection. All methods were carried out in accordance with relevant guidelines and regulations, and all participants provided informed consent. The survey material and description of the data collection procedure can be accessed in Terry et al.’s^56^ OSF linkhttps://osf.io/mhg94/. The current project was pre-registered, following suggestions for pre-registration of secondary data analyses^52^. We did not discuss in this manuscript: Aims 1 and 4, where we first established whether these measures of mathematics and statistics anxiety are valid for the neurodivergent population, using item response theory (Aim 1), and where we explore the question of whether we can discriminate between neurotypical and neurodivergent participants based on anxiety profiles using latent class analysis (Aim 4), as these were not the focus of the manuscript. In addition, we planned to use a general linear mixed model (GLMM) originally to assess Aims 2 and 3, as outlined in the pre-registration. However, as only one response per participant was available, the hierarchical structure required for GLMM was not present in our data. Therefore, GLMM was not an appropriate method to investigate our research question. Therefore, we deviated from the pre-registered analysis plan and employed a general linear multiple regression.

### Participants and procedure

The study was approved by the Ethics Committees of various universities (see OSF). We conducted a secondary analysis of the SMARVUS data^56^, that involved 12,570 participants. The survey was given to undergraduate students who had statistics as a core module, but who were not majoring in subjects where mathematics or statistics were a main part of the programme (e.g., excluding physics and engineering but including psychology and business). We followed the same inclusion criteria as Terry et al.^56^ where they excluded those who withdrew before the first block of measurement scale, duplicates, and participants from courses typically associated with mathematics (e.g. physics, engineering and data science). We removed 70 participants’ data: 37 neurodivergent individuals who responded, ‘other undiagnosed’, 30 who responded ‘other (unspecified)’, 3 ‘unsure’. 2,692 participants did not respond to this item. We removed 1221 cases with missing data on age, gender, education and country of origin. We also removed data from four countries (Ghana, Philippines, Saudi Arabia, and Slovenia) as no neurodivergent sample from these countries took part in the study. These steps were taken to ensure that the neurotypical and neurodivergent groups could be matched on age, gender, level of education and country of residence.

After matching groups on age, gender, level of education and country of residence, using data from 8453 participants, we had a final sample of 1383 participants, which included 679 neurotypical and 704 neurodivergent individuals. Chi-square tests for independence for age [*X*^2^ (2, *N* = 1,387) = 0.61, *p* = 0.738], gender [*X*^2^ (1, *N* = 1,387) = 0.15, *p* = 0.702], education [*X*^2^ (4, *N* = 1,387) = 0.48, *p* = 0.976] and country of residence [*X*^2^ (32, *N* = 1,387) = 1.79, *p* > 0.999], indicated that the groups were very well-matched on these variables. For further details about the population characteristics, see Supplementary materials (Tables S1 and S2).

### General procedures for the tests

Each participant completed all components of the study. Participants were assessed on several measures of anxiety and attitudes, and the CRT, which are described in detail in Terry et al.^56^ (see Table 7).

**Table 7:** Individual differences measures.

Tests	Administration	Measures	*ω/α*
Anxiety and social cognition			
Uncertainty scale-short form (IUS-SF)^58^	Score from1 [Not At All Characteristic of Me] to 5 [Entirely Characteristic of Me] for uncertainty (12 items)	Intolerance of uncertainty	*ω* (ML) = 0.906 *ω* (HA) = 0.905 α = 0.905
Brief fear of negative evaluation scale—straightforward (BNFE-S)^59,60^	Score from 1 [Not At All Characteristic of Me] to 5 [Entirely Characteristic of Me] for negative evaluation (8 items)	Fear of negative evaluation	*ω* (ML) = 0.949 *ω* (HA) = 0.949 α = 0.949
Liebowitz social anxiety scale (LSAS-SR)^66,67^	Score from 0 [Not At All] to 3 [Very Much So] for social anxiety (12 items) and performance anxiety (12 items). Participants indicate how anxious they would feel in each situation	Social Anxiety and performance anxiety	*ω* (ML) = 0.933 *ω* (HA) = 0.932 α = 0.932
Anxiety and performance			
Creativity anxiety scale^51^	Score from 0 [Not at all] to 4 [Very Much So] for creativity anxiety (8 items). Participants are asked to indicate how anxious they would feel in each situation	(Non-) creativity anxiety	*ω* (ML) = 0.914 *ω* (HA) = 0.904 α = 0.914
State-trait inventory for cognitive and somatic anxiety (STICSA)^69^	Score from 1 [None] to 4 [Very Much So] for cognitive (10 items) and somatic (11 items) symptoms. Participants are asked to indicate which item is true	Cognitive and somatic anxiety	*ω* (ML) = 0.930 *ω* (HA) = 0.929 α = 0.930
Test anxiety (R-TAS)^70^	Score from 1 [Almost Never] to 4 [Almost Always] for worry (6 items), tension (5 items), test-irrelevant thinking (4 items) and bodily symptoms (5 items). Participants rate each item in terms of how they feel when taking tests in general	Test anxiety	*ω* (ML) = 0.945 *ω* (HA) = 0.944 α = 0.945
Cognitive abilities and motivation			
Cognitive reflection test (CRT)^24,54^	Responses receive a score of 1 (Correct) or 0 (Incorrect) for analytic thinking (3 items), and a score of 1 (typical intuitive response) or 0 (any other response) for intuitive thinking	Analytic thinking	*ω* (ML) = 0.682 *ω* (HA) = 0.682 α = 0.678
Cognitive reflection test (CRT)-intuitive responses^24,54^	Responses receive a score of 1 (Correct) or 0 (Incorrect) for analytic thinking (3 items), and a score of 1 (typical intuitive response) or 0 (any other response) for intuitive thinking	Intuitive thinking	*ω* (ML) = 0.678 *ω* (HA) = 0.641 α = 0.637
Attitude towards mathematics survey persistence subscale (ATMS)^71^	Score from 1 [Strongly Disagree] to 5 [Strongly Agree] for academic persistence (8 items)	Academic persistence	*ω* (ML) = 0.747 *ω* (HA) = 0.737 α = 0.742
New general self-efficacy scale (NGSE)^72^	Score from 1 [Strongly Disagree] to 5 [Strongly Agree] for self-efficacy (8 items)	Self-efficacy	*ω* (ML) = 0.894 *ω* (HA) = 0.894 α = 0.894
Statistics and mathematics anxiety			
Statistics anxiety rating scale (STARS)^56,57^	The three anxiety subscales were used: test and class anxiety (8 items), interpretation anxiety (11 items), and fear of asking for help (4 items). Participants are asked to indicate how much anxiety they feel in those situations ranging from 1 [No Anxiety] to 5 [Very Much Anxiety]	Statistic anxiety	STARS *ω* (ML) = 0.951 *ω* (HA) = 0.950 α = 0.951 STARS-M *ω* (ML) = 0.943 *ω* (HA) = 0.941 α = 0.943
Revised maths anxiety rating scale (R-MARS)^74^	Three subscales which measure mathematics test anxiety (15 items), numerical task anxiety (5 items), and mathematics course anxiety (5 items). Participants are asked to indicate how much anxiety they feel in those situations ranging from 1 [No Anxiety] to 5 [Very Much Anxiety]	Maths anxiety	*ω* (ML) = 0.955 *ω* (HA) = 0.952 α = 0.954

All alpha / omega values based on analysis of the current data set. *ω* (ML) = omega estimated via forced single-factor maximum-likelihood factor analysis; *ω* (HA) = omega estimated via approximate and closed-form solution as per Hancock and An (2020); α = Cronbach’s alpha.

Across measures, omega and alpha measures were more-than-acceptable (Table 7). Values for the CRT^16,70^ were in line with previous studies^47^.

Participants were also given a demographic questionnaire that included questions of age (in years), gender identity, ethnicity, and whether they have been identified with a specific learning disability/neurodivergence, such as dyslexia or dyscalculia. Additionally, participants were asked to complete their course details, current year of study, whether they study other maths-based modules/courses on their degree; their highest level of pre-university maths education (e.g., GCSE or A-level or international equivalents).

### Missing values

For the analyses, we recorded gender as a dichotomous variable (male/female). The data originally had three categories (male, female, and other). “Other” was recorded as missing value (*n* = 151). We removed 1221 cases with any missing data on age, gender, education and country of origin. Data labelled as “Other” for gender was excluded during the matching process. Although we acknowledge the disadvantages of this approach, we decided to do this as the term “other” is ambiguous and could represent any gender. Additionally, we wanted to include gender as a covariate in some of our analyses, which is only possible with binary coding. After removing the missing data, the two groups (neurotypical and neurodivergent) were matched based on age, gender, education, and country of origin. After initially matching groups, we further located the missing values in the self-report measures. Data of 6 participants was removed because they did not complete five or more (> 40%) out of the 12 measures. Further, five participants (1 for statistics anxiety, 1 for mathematics anxiety, 2 for cognitive and somatic anxiety, and 1 for social anxiety) had partial missing data (> 10%). In these cases, the total score for a specific measure (e.g. social, mathematics, cognitive and somatic anxiety) was coded as missing. Furthermore, 5 participants (1 for statistics anxiety, 2 for mathematics anxiety, 1 for cognitive and somatic anxiety, and 1 for intolerance of uncertainty) had less than 10% missing data on that measure. In this case, the missing values were replaced with the participant’s modal response on that particular measure. Little’s^71^ test for Missing Completely at Random (MCAR; *χ*^2^ (40) = 67.56, *p* = 0.004) indicated that values were not missing at random. The missing data was addressed using multiple imputations to reduce bias and increase the validity of statistical inference^72^. Five imputed datasets were generated, using the pooled estimates to perform the correlation and hierarchical regression analyses. However, there was no significant difference between the results of the imputed and the original datasets, indicating that multiple imputations did not significantly change the findings. The results of the imputed data can be found in the supplementary materials (Tables 2, 3 and 4a) and on the OSF page.

### Notes

^I^ To refer to neurodivergent people, we are using identity-first language throughout the manuscript, as it seems to reflect the preferences of this community better (see^73^). However, we are also aware of arguments against using identity-first language^73^ and that preferences may vary from person to person.

## Supplementary Information

Below is the link to the electronic supplementary material.

Supplementary Material 1

## Data Availability

The data and code used in the analyses reported can be accessed via the Open Science Framework. (please [https://osf.io/ypa8z/]
