# Supplementary material for: Academic anxiety and cognitive reflection in neurodivergence based on evidence from a large international sample
Source: Sci Rep. 2025 Oct 27;15:37522. doi: 10.1038/s41598-025-21504-6 (PMC12559732; doi:10.1038/s41598-025-21504-6)
Supplement: Supplementary file 1 — Supplementary Material 1 [file 41598_2025_21504_MOESM1_ESM.docx]

# Supplementary materials

***Table S1.***

*Description of the study population in terms of the demographic variables used for matching the neurotypical (NT) and neurodivergent (ND) samples (i.e., age, gender, level of education, and country of residence)*

|  | ***N (NT)*** | ***N (ND)*** | ***Total N*** | ***Chi-squared statistics*** |
| --- | --- | --- | --- | --- |
| **Age** |  |  |  | *X*^2^ (2) =.61 , *p* =.738 |
| 18-21 | 417 | 421 | 838 |  |
| 22-25 | 200 | 211 | 411 |  |
| 26+ | 62 | 72 | 134 |  |
| **Gender** |  |  |  | *X*^2^ (1) =.15, *p* =.702 |
| Male | 139 | 150 | 289 |  |
| Female | 540 | 554 | 1,094 |  |
| **Education** |  |  |  | *X*^2^ (4) =0.48, *p* = .976 |
| 1^st^ year | 355 | 360 | 715 |  |
| 2^st^ year | 201 | 208 | 409 |  |
| 3^rd^ yea | 88 | 99 | 187 |  |
| 4^th^ year | 31 | 32 | 63 |  |
| 5^th^ year | 4 | 5 | 9 |  |
| **Country of Residence** |  |  |  | *X*^2^ (32) =1.79, *p* > .999 |
| Australia | 23 | 24 | 47 |  |
| Austria | 7 | 7 | 14 |  |
| Belgium | 20 | 21 | 41 |  |
| Brazil | 3 | 5 | 8 |  |
| Canada | 61 | 61 | 122 |  |
| Chile | 12 | 15 | 27 |  |
| China | 2 | 2 | 4 |  |
| Colombia | 3 | 3 | 6 |  |
| Egypt | 71 | 72 | 143 |  |
| England | 208 | 208 | 416 |  |
| Estonia | 4 | 4 | 8 |  |
| France | 7 | 7 | 14 |  |
| Germany | 20 | 20 | 40 |  |
| Greece | 3 | 3 | 6 |  |
| Hungary | 6 | 6 | 12 |  |
| India | 1 | 1 | 2 |  |
| Indonesia | 7 | 7 | 14 |  |
| Israel | 98 | 105 | 203 |  |
| Italy | 9 | 10 | 19 |  |
| Malaysia | 4 | 5 | 9 |  |
| Netherlands | 44 | 45 | 89 |  |
| Nigeria | 1 | 1 | 2 |  |
| Northern Ireland | 8 | 9 | 17 |  |
| Poland | 6 | 7 | 13 |  |
| Republic of Ireland | 3 | 3 | 6 |  |
| Romania | 3 | 3 | 6 |  |
| Scotland | 12 | 14 | 26 |  |
| Serbia | 1 | 1 | 2 |  |
| Slovakia | 2 | 2 | 4 |  |
| Spain | 13 | 12 | 25 |  |
| Turkey | 5 | 5 | 10 |  |
| Ukraine | 1 | 1 | 2 |  |
| USA | 11 | 15 | 26 |  |

***Note.*** *X^2^ = chi-square; all Ns = 1,383; NT = neurotypical; ND = neurodivergent.*

**Table S2.**

*Frequency of different conditions within the neurodivergent sample*

| **Identification** | ***N*** |
| --- | --- |
| ADHD/ADD | 293 |
| ADHD/ADD, Dyscalculia | 3 |
| ADHD/ADD, Dysgraphia/Dysorthography | 5 |
| ADHD/ADD, Dyslexia | 1 |
| ADHD/ADD, Other - Autism Spectrum Disorder | 3 |
| ADHD/ADD, Other - Not an SpLD | 1 |
| ADHD/ADD, Other - Redacted | 1 |
| Dyscalculia | 57 |
| Dyscalculia, Dysgraphia/Dysorthography | 1 |
| Dyscalculia, Other - Unspecified | 1 |
| Dysgraphia/Dysorthography | 11 |
| Dyslexia | 181 |
| Dyslexia, ADHD/ADD | 16 |
| Dyslexia, ADHD/ADD, Dyscalculia, Dysgraphia/Dysorthography | 1 |
| Dyslexia, ADHD/ADD, Dysgraphia/Dysorthography | 2 |
| Dyslexia, Dyscalculia | 19 |
| Dyslexia, Dyscalculia, Dysgraphia/Dysorthography | 2 |
| Dyslexia, Dyscalculia, Dysgraphia/Dysorthography, Dyspraxia | 1 |
| Dyslexia, Dysgraphia/Dysorthography | 13 |
| Dyslexia, Dyspraxia | 12 |
| Dyslexia, Dyspraxia, ADHD/ADD | 6 |
| Dyslexia, Dyspraxia, Other - Redacted | 1 |
| Dyslexia, Other - Not an SpLD | 1 |
| Dyslexia, Other - Redacted | 3 |
| Dyspraxia | 14 |
| Dyspraxia, Dysgraphia/Dysorthography | 1 |
| Other - Autism Spectrum Disorder | 16 |
| Other - Not an SpLD | 14 |
| Other - Redacted | 24 |
| **Total** | 704 |

**Table S3.**

*Hierarchical regression predicting CRT performance (intuitive responses) by age, gender, anxiety and attitude measures*

| **Dependent variable:** CRT intuitive response | | | | | | | | | | |  |
| --- | --- | --- | --- | --- | --- | --- | --- | --- | --- | --- | --- |
|  | **Neurotypical group** | | | | | | **Neurodivergent group** | | | | |
|  | ***b*** | ***beta*** | ***t*** | ***p*** | ***Adj R^2^*** | ***b*** | ***beta*** | ***t*** | ***p*** | ***Adj R^2^*** | |
| **Step 1** |  |  |  |  | .044* |  |  |  |  | *.*059* | |
| Age | -0.01 | -0.01 | -0.23 | .816 |  | <0.01 | 0.03 | 0.00 | 1.00 |  | |
| Gender | 0.60 | 0.22 | 5.71 | <.001** |  | 0.69 | -0.23 | 6.74 | <.001** |  | |
| **Step 2** |  |  |  |  | .141* |  |  |  |  | .149* | |
| Age | -0.05 | -0.03 | -0.83 | .409 |  | -0.01 | 0.02 | -0.10 | .924 |  | |
| Gender | 0.52 | 0.19 | 4.95 | <.001** |  | 0.53 | -0.17 | 5.32 | <.001** |  | |
| Statistics Anxiety | <0.01 | -0.03 | -0.50 | .619 |  | <0.01 | -0.10 | 1.04 | .299 |  | |
| Mathematics Anxiety | 0.02 | 0.28 | 4.88 | <.001** |  | 0.01 | -0.16 | 3.70 | <.001** |  | |
| Test Anxiety | <0.01 | 0.03 | 0.60 | .550 |  | 0.01 | -0.09 | 1.56 | .120 |  | |
| Cognitive and Somatic Anxiety | <0.01 | 0.23 | 0.36 | .719 |  | <0.01 | 0-.01 | 0.47 | .640 |  | |
| Fear of Negative Evaluation | -0.03 | -0.03 | -4.68 | <.001** |  | -0.02 | 0.19 | -2.67 | .008** |  | |
| Social Anxiety | <0.01 | -0.02 | -0.35 | .619 |  | <0.01 | 0.03 | -0.48 | .632 |  | |
| Creativity Anxiety | 0.01 | -0.12 | -2.70 | .147 |  | 0.01 | -0.14 | 1.79 | .074 |  | |
| Intolerance of Uncertainty | 0.01 | -0.05 | -0.94 | .191 |  | -0.01 | 0.02 | -1.49 | .138 |  | |
| General Self-Efficacy | 0.02 | -0.15 | -3.81 | .003** |  | 0.01 | -0.03 | 1.75 | .080 |  | |
| Attitude Towards Mathematics | -0.01 | 0.08 | 2.06 | .186 |  | <0.01 | 0.05 | 0.07 | .944 |  | |

**Note.****p*<.05 ***p*<.001; *b*= unstandardized regression weights, *beta*= standardized regression weights. CRT = Cognitive Reflection Test.

## The moderating effect of neurodivergent status on predictors of performance on the CRT performance (intuitive responses)

To statistically assess the presence of any group differences, we conducted an additional hierarchical regression analysis for the combined neurodivergent and neurotypical sample, incorporating interaction terms for neurodivergent status. This enabled us to statistically test whether measures exhibited different associations with CRT performance (intuitive responses) across the two groups (Table S4). Age (a continuous measure) and gender (coded as -1= male, 1= female) were included in Block 1. In Block 2, all other measures were entered (main effects), whereas in Block 3, interaction terms between each predictor and neurodivergent (ND) status were added.

**Table S4**

*Hierarchical regression predicting CRT performance (intuitive responses); main effects and interactions with neurodivergent status*

|  | **Step 1** | | | | **Step 2** | | | | **Step 3** | | | |
| --- | --- | --- | --- | --- | --- | --- | --- | --- | --- | --- | --- | --- |
|  | ***b*** | ***beta*** | ***t*** | ***p*** | ***b*** | ***beta*** | ***t*** | ***p*** | ***b*** | ***beta*** | ***t*** | ***p*** |
|  |  |  |  |  |  |  |  |  |  |  |  |  |
| Age | -0.01 | 0.00 | -0.17 | .868 | -0.02 | -0.01 | -0.52 | .605 | -0.03 | -0.02 | -0.63 | .528 |
| Gender | 0.65 | 0.23 | 8.82 | <.001** | 0.53 | 0.19 | 7.34 | <.001** | 0.53 | 0.19 | 7.27 | <.001** |
| Statistics Anxiety |  |  |  |  | 0.00 | 0.02 | 0.48 | .633 | 0.00 | -0.03 | -0.52 | .602 |
| Mathematics Anxiety |  |  |  |  | 0.01 | 0.24 | 5.86 | <.001** | 0.01 | 0.29 | 4.86 | <.001** |
| Test Anxiety |  |  |  |  | 0.00 | 0.06 | 1.61 | .107 | 0.00 | 0.04 | 0.64 | .525 |
| Creativity Anxiety |  |  |  |  | 0.01 | 0.08 | 2.40 | .017* | 0.01 | 0.07 | 1.50 | .134 |
| Fear of Negative Evaluation |  |  |  |  | -0.02 | -0.18 | -5.19 | <.001** | -0.03 | -0.23 | -4.66 | <.001** |
| Social Anxiety |  |  |  |  | 0.00 | 0.00 | -0.08 | .934 | 0.00 | 0.03 | 0.49 | .627 |
| Cognitive and Somatic Anxiety |  |  |  |  | 0.00 | 0.02 | 0.63 | .527 | 0.00 | 0.02 | 0.39 | .694 |
| Intolerance of Uncertainty |  |  |  |  | 0.00 | -0.01 | -0.22 | .830 | 0.01 | 0.06 | 1.28 | .199 |
| General Self-Efficacy |  |  |  |  | 0.02 | 0.09 | 3.31 | <.001** | 0.02 | 0.13 | 3.25 | .001** |
| Attitude Towards Mathematics |  |  |  |  | -0.01 | -0.03 | -0.89 | .372 | -0.01 | -0.06 | -1.52 | .130 |
| Statistics Anxiety*ND |  |  |  |  |  |  |  |  | 0.01 | 0.17 | 1.11 | .267 |
| Mathematics Anxiety*ND |  |  |  |  |  |  |  |  | 0.00 | -0.14 | -0.93 | .351 |
| Test Anxiety*ND |  |  |  |  |  |  |  |  | 0.00 | 0.10 | 0.64 | .524 |
| Creativity Anxiety *ND |  |  |  |  |  |  |  |  | 0.00 | 0.03 | 0.28 | .780 |
| Fear of Negative Evaluation*ND |  |  |  |  |  |  |  |  | 0.01 | 0.15 | 1.37 | .170 |
| Social Anxiety*ND |  |  |  |  |  |  |  |  | 0.00 | -0.11 | -0.72 | .473 |
| Cognitive and Somatic Anxiety *ND |  |  |  |  |  |  |  |  | 0.00 | 0.00 | 0.02 | .985 |
| Intolerance of Uncertainty*ND |  |  |  |  |  |  |  |  | -0.01 | -0.25 | -1.96 | .050 |
| General Self-Efficacy*ND |  |  |  |  |  |  |  |  | -0.01 | -0.16 | -1.29 | .196 |
| Attitude Towards Mathematics*ND |  |  |  |  |  |  |  |  | 0.01 | 0.16 | 1.34 | .179 |
| ***Adj R^2^ Adj R^2^*** | .053** | | | | .146** | | | | .147 | | | |

**Note .****p*<.05 ***p*<.001; *b* = unstandardized regression weights, *beta* = standardized regression weights; CRT = Cognitive Reflection Test

The results indicated that mathematics anxiety, self-efficacy and fear of negative evaluation were significantly more strongly associated with CRT intuitive responses in the neurotypical than in the neurodivergent group. Nevertheless, mathematics anxiety and fear of negative evaluation were significant predictors of CRT performance (intuitive responses) in both groups, as indicated by the separate regression analyses, whereas self-efficacy only had a significant effect in the case of the neurotypical group. However, no interaction was associated with significant main effects in either group.

# **Supplementary analyses relating to specific neurodivergent conditions**

In the dataset, we had five subgroups among the neurodivergent population with no co-occurrences (i.e., ADHD, autism, dyscalculia, dyslexia, dyspraxia). We matched each subgroup with a subgroup of the neurotypical group, based on age, gender, education level, and country of residence, using propensity score matching (many-to-one method). After matching each subgroup separately with a subgroup of the neurotypical sample, we conducted *t* tests to find out about group differences in our study variables.

To further investigate predictors of group membership, we performed hierarchical regression analyses for three subgroups (dyslexia, ADHD, and dyscalculia) where sample sizes were sufficient for the purposes of such analyses. In the regression model, we used the neurotypical/neurodivergent category as the outcome variable (0 = neurotypical; 1 = neurodivergent), with all other measures as predictors, and added age and gender (-1 = male; 1 = female) as covariates.

Given that some variables (especially the anxiety measures) were highly correlated, we also computed multicollinearity statistics. The analysis showed that the VIF value for each predictor was acceptable (VIF < 3). In addition, the models met the conditions of independence of residuals.

## Analyses relating to dyslexia

178 neurotypical participants were matched with 181 dyslexic individuals on age, gender, country of residence, and education level. Population characteristics, descriptive statistics and independent *t* test results comparing the neurotypical and dyslexic individuals on all measures are presented in Table S5 and S6.

**Table S5.**

*Description of the study population in relation to the demographic variables used for matching the groups (age, gender, level of education, and country of residence)*

|  | **NT** | **Dyslexia** | **Total** | **Statistics** |
| --- | --- | --- | --- | --- |
| **Age** |  |  |  | *X*^2^ (2) =.012, *p* = .941 |
| 18-21 | 126 | 126 | 252 |  |
| 22-25 | 38 | 39 | 77 |  |
| 26+ | 14 | 16 | 30 |  |
| **Gender** |  |  |  | *X*^2^ (1) =0.004, *p* = .947 |
| Male | 27 | 27 | 54 |  |
| Female | 151 | 154 | 305 |  |
| **Education** |  |  |  | *X*^2^ (3) =0.08, *p* = .995 |
| 1^st^ year | 84 | 85 | 169 |  |
| 2^st^ year | 67 | 67 | 134 |  |
| 3^rd^ yea | 20 | 22 | 42 |  |
| 4^th^ year | 7 | 7 | 14 |  |
| **Country of Residence** |  |  |  | *X*^2^ (21) =0.17, *p* > .999 |
| Australia | 8 | 8 | 16 |  |
| Austria | 2 | 2 | 4 |  |
| Belgium | 6 | 6 | 12 |  |
| Canada | 8 | 8 | 16 |  |
| Chile | 1 | 1 | 2 |  |
| Egypt | 10 | 11 | 21 |  |
| England | 96 | 96 | 192 |  |
| France | 2 | 2 | 4 |  |
| Greece | 1 | 1 | 2 |  |
| Hungary | 1 | 1 | 2 |  |
| India | 1 | 1 | 2 |  |
| Indonesia | 1 | 1 | 2 |  |
| Italy | 2 | 2 | 4 |  |
| Netherlands | 13 | 14 | 27 |  |
| Northern Ireland | 4 | 5 | 9 |  |
| Poland | 4 | 4 | 8 |  |
| Republic of Ireland | 2 | 2 | 4 |  |
| Romania | 1 | 1 | 2 |  |
| Scotland | 9 | 9 | 18 |  |
| Slovakia | 1 | 1 | 2 |  |
| Spain | 3 | 3 | 6 |  |
| USA | 2 | 2 | 4 |  |

**Note.** *X*^2^ *= chi-square; all Ns =* 359

**Table S6.**

*Independent t tests comparing the neurotypical (n = 178) and dyslexic (n = 181)* *individuals*

|  | **Neurotypical** | | **Dyslexia** | |  |  |
| --- | --- | --- | --- | --- | --- | --- |
| **Measures** | *M (SD)* | *Range* | *M (SD)* | *Range* | *p* | *Cohen’s d* |
| Statistics Anxiety | 71.28 (19.86) | 23-115 | 71.34 (20.14) | 29-115 | .975 | <0.01 |
| Mathematics Anxiety | 75.71 (20.42) | 24-117 | 74.48 (21.08) | 29-119 | .671 | 0.04 |
| Cognitive & Somatic Anxiety | 46.90 (13.77) | 22-82 | 49.92 (14.24) | 21-82 | .042* | 0.22 |
| Test Anxiety | 62.79 (16.57) | 25-99 | 62.72 (15.97) | 27-99 | .968 | <0.01 |
| Fear of Negative Evaluation | 26.51 (9.24) | 8-40 | 25.72 (9.05) | 8-40 | .415 | 0.09 |
| Social Anxiety | 60.24 (14.84) | 27-92 | 58.69 (15.22) | 26-93 | .327 | 0.10 |
| Creativity Anxiety | 42.44 (12.62) | 16-78 | 42.03 (11.13) | 17-80 | .744 | 0.03 |
| Intolerance of Uncertainty | 34.96 (11.15) | 12-60 | 35.33 (10.52) | 15-60 | .746 | 0.03 |
| General Self-Efficacy | 28.06 (5.02) | 14-40 | 27.08 (5.38) | 9-38 | .075 | 0.19 |
| Attitude Towards Mathematics | 24.29 (4.85) | 11-35 | 23.77 (4.23) | 11-34 | .286 | 0.11 |
| CRT (Deliberative response) | 0.62 (0.87) | 0-3 | 1.00 (1.16) | 0-3 | <.001** | 0.37 |
| CRT (Intuitive response) | 2.03 (0.98) | 0-3 | 1.68 (1.15) | 0-3 | .002** | 0.33 |

**Note.** **p* < .05, ***p* < .01; CRT = Cognitive Reflection Test

To further investigate the predictors of group membership, we conducted a hierarchical regression analysis. In the regression model, we used neurotypical/dyslexic as the outcome variable (0 = neurotypical; 1 = dyslexic), with all other measures as predictors, and added age and gender (-1 = male; 1 = female) as covariates. Given that some variables (especially the anxiety measures) were highly correlated, we also computed multicollinearity statistics. The analysis showed that the VIF value for each predictor was acceptable (VIF < 3). In addition, the model met the conditions of independence of residuals. As a result, it was possible to estimate regression weights with confidence.

A significant effect was observed in the model (*F* (13, 344) = 2.60, *p =* .002, *η*^2^ = .055). The analysis showed that after controlling for the effects of age and gender, higher cognitive and somatic anxiety and higher levels of cognitive reflection increased the probability of being dyslexic. The remaining variables were non-significant predictors (see Table S7).

**Table S7.**

*Hierarchical regression predicting group membership by demographics, anxiety and attitude measures, and the CRT*

| ***Dependent variable: Dyslexia status*** | | | | | |
| --- | --- | --- | --- | --- | --- |
|  | ***b*** | ***beta*** | ***t*** | ***p*** | ***Adj R^2^*** |
| **Step 1** |  |  |  |  | .005 |
| Age | 0.01 | 0.02 | 0.29 | .775 |  |
| Gender | 0.00 | -0.00 | -0.05 | .957 |  |
| **Step 2** |  |  |  |  | .055 |
| Age | 0.02 | 0.03 | 0.54 | .587 |  |
| Gender | 0.02 | 0.03 | 0.64 | .524 |  |
| Statistics Anxiety | 0.00 | 0.09 | 1.02 | .310 |  |
| Mathematics Anxiety | 0.00 | -0.01 | -0.13 | .895 |  |
| Cognitive and Somatic Anxiety | 0.01 | 0.26 | 3.18 | .002** |  |
| Test Anxiety | 0.00 | -0.07 | -0.81 | .416 |  |
| Fear of Negative Evaluation | -0.01 | -0.14 | -1.87 | .062 |  |
| Social Anxiety | 0.00 | -0.11 | -1.45 | .148 |  |
| Creativity Anxiety | 0.00 | -0.05 | -0.72 | .473 |  |
| Intolerance of Uncertainty | 0.00 | 0.03 | 0.34 | .731 |  |
| General Self-Efficacy | -0.01 | -0.05 | -0.93 | .355 |  |
| Attitude Towards Mathematics | -0.01 | -0.09 | -1.49 | .136 |  |
| Cognitive Reflection Test | 0.10 | 0.21 | 3.61 | <.001** |  |

**Note** **p* < .05, ***p* < .01; *b*= unstandardized regression weights, *beta*=standardised regression weights; CRT = Cognitive Reflection Test.

##

## Analyses relating to ADHD

283 neurotypical participants were matched with 293 ADHD individuals on age, gender, country of residence and education. Six missing values were found across self-report measures. However, Little’s (1988) test for Missing Completely at Random (MCAR) (*χ*^2^ (21)=14.12, *p*=.864), showed that data was missing at random and no imputation of missing values was required. Population characteristics, descriptive statistics and independent *t* test results comparing the neurotypical and ADHDers on all measures are presented in Table S8 and S9.

**Table S8**

*Description of the study population in relation to demographic variables used for matching (age, gender, level of education and country of residence)*

|  | **NT (n)** | **ADHD/ADD (n)** | **Total** | **Statistics** |
| --- | --- | --- | --- | --- |
| **Age** |  |  |  | *X*^2^ (2) = 0.22, *p* =.897 |
| 18-21 | 144 | 146 | 290 |  |
| 22-25 | 115 | 119 | 234 |  |
| 26+ | 24 | 28 | 52 |  |
| **Gender** |  |  |  | *X*^2^ (1) = 0.07, p =.790 |
| Male | 64 | 69 | 133 |  |
| Female | 219 | 224 | 443 |  |
| **Education** |  |  |  | *X*^2^ (4) = 0.29, p =.990 |
| 1^st^ year | 164 | 167 | 331 |  |
| 2^st^ year | 59 | 60 | 119 |  |
| 3^rd^ yea | 37 | 42 | 79 |  |
| 4^th^ year | 19 | 19 | 38 |  |
| 5^th^ year | 4 | 5 | 9 |  |
| **Country of Residence** |  |  |  | *X*^2^ (25) = 1.01, p > .999 |
| Australia | 12 | 12 | 24 |  |
| Austria | 2 | 2 | 4 |  |
| Belgium | 7 | 8 | 15 |  |
| Brazil | 3 | 5 | 8 |  |
| Canada | 42 | 42 | 84 |  |
| Chile | 11 | 13 | 24 |  |
| China | 2 | 2 | 4 |  |
| Colombia | 3 | 3 | 6 |  |
| Egypt | 26 | 26 | 52 |  |
| England | 27 | 27 | 54 |  |
| Estonia | 1 | 1 | 2 |  |
| France | 2 | 2 | 4 |  |
| Germany | 4 | 4 | 8 |  |
| Greece | 1 | 1 | 2 |  |
| Hungary | 2 | 2 | 4 |  |
| Indonesia | 5 | 5 | 10 |  |
| Israel | 85 | 87 | 172 |  |
| Italy | 1 | 1 | 2 |  |
| Malaysia | 4 | 4 | 8 |  |
| Netherlands | 23 | 23 | 46 |  |
| Romania | 1 | 1 | 2 |  |
| Scotland | 2 | 3 | 5 |  |
| Serbia | 1 | 1 | 2 |  |
| Spain | 3 | 3 | 6 |  |
| Turkey | 5 | 5 | 10 |  |
| USA | 8 | 10 | 18 |  |

**Note.** *X*^2^ *= chi-square; all Ns* = 576

**Table S9.**

*Independent t tests of all measures for the Neurotypical (n = 283) and ADHD (n = 293) group*

|  | **Neurotypical** | | **ADHD** | |  |  |
| --- | --- | --- | --- | --- | --- | --- |
| **Measures** | *M (SD)* | *Range* | *M (SD)* | *Range* | *p* | *Cohen’s d* |
| Statistics Anxiety | 67.06 (20.10) | 23-114 | 65.39 (21.50) | 23-115 | .337 | 0.08 |
| Mathematics Anxiety | 71.76 (21.37) | 24-117 | 72.23 (22.92) | 24-120 | .799 | 0.02 |
| Cognitive and Somatic Anxiety | 42.58 (13.45) | 21-82 | 47.29 (14.36) | 21-84 | <.001** | 0.34 |
| Test Anxiety | 57.52 (17.50) | 25-99 | 61.74 (17.42) | 25-100 | .004** | 0.24 |
| Fear of Negative Evaluation | 24.25 (9.26) | 8-40 | 25.35 (9.04) | 8-40 | .148 | 0.12 |
| Social Anxiety | 54.39 (15.51) | 24-94 | 54.66 (14.13) | 25-96 | .832 | 0.02 |
| Creativity Anxiety | 40.85 (12.73) | 16-78 | 41.06 (12.02) | 16-80 | .839 | 0.02 |
| Intolerance of Uncertainty | 34.07(10.43) | 12-60 | 34.61 (10.28) | 12-60 | .534 | 0.05 |
| General Self-Efficacy | 30.29 (5.26) | 16-40 | 29.23 (5.90) | 8-40 | .023* | 0.19 |
| Attitude Towards Mathematics | 24.89 (4.48) | 11-35 | 24.68 (5.13) | 10-35 | .600 | 0.04 |
| CRT (Deliberative response) | 0.94(1.08) | 0-3 | 1.00 (1.11) | 0-3 | .115 | 0.13 |
| CRT (Intuitive response) | 1.78(1.07) | 0-3 | 1.63(1.14) | 0-3 | .103 | 0.14 |

**Note.** **p* < .05, ***p* < .01; CRT = Cognitive Reflection Test.

To further investigate the predictors of group membership, we conducted a hierarchical regression analysis. In the regression model, we used neurotypical/ADHD as the outcome variable (0 = neurotypical; 1 = ADHD), with all other measures as predictors, and added age and gender (-1 = male; 1 = female) as covariates. Given that some variables (especially the anxiety measures) were highly correlated, we also computed multicollinearity statistics. The analysis showed that the VIF value for each predictor was acceptable (VIF < 3). In addition, the model met the conditions of independence of residuals. As a result, it was possible to estimate regression weights with confidence.

A significant effect was observed in the model (*F* (13, 559) = 3.49, *p* < .001, *η*^2^ = .075). The analysis showed that after controlling for the effects of age and gender, ADHD status was associated with higher cognitive and somatic anxiety and test anxiety, and lower statistics and social anxiety. All remaining variables were non-significant.

**Table S10.**

*Hierarchical regression predicting group membership by demographics, anxiety measures, and the CRT*

| **Dependent variable: ADHD status** | | | | | |
| --- | --- | --- | --- | --- | --- |
|  | ***b*** | ***beta*** | ***t*** | ***p*** | ***Adj R^2^*** |
| **Step 1** |  |  |  |  | .003 |
| Age | 0.01 | 0.01 | 0.32 | .745 |  |
| Gender | -0.01 | -0.01 | -0.27 | .785 |  |
| **Step 2** |  |  |  |  | .054 |
| Age | 0.05 | 0.07 | 1.58 | .114 |  |
| Gender | <0.01 | 0.00 | -0.01 | .989 |  |
| Statistics Anxiety | <0.01 | -0.14 | -2.08 | .038* |  |
| Mathematics Anxiety | <0.01 | 0.03 | 0.53 | .599 |  |
| Cognitive and Somatic Anxiety | 0.01 | 0.26 | 3.91 | <.001** |  |
| Test Anxiety | <0.01 | 0.14 | 2.20 | .028* |  |
| Fear of Negative Evaluation | <0.01 | -0.01 | -.21 | .832 |  |
| Social Anxiety | <0.01 | -0.12 | -2.00 | .046* |  |
| Creativity Anxiety | <0.01 | -0.04 | -0.76 | .449 |  |
| Intolerance of Uncertainty | <0.01 | -0.05 | -0.90 | .371 |  |
| General Self-Efficacy | -0.01 | -0.08 | -1.76 | .078 |  |
| Attitude Towards Mathematics | <0.01 | 0.01 | 0.27 | .788 |  |
| Cognitive Reflection Test | 0.03 | 0.06 | 1.26 | .209 |  |

**Note.** *b* = unstandardized regression weights, *beta* = standardized regression weights; CRT = Cognitive Reflection Test

## Analyses relating to dyscalculia

56 neurotypical participants were matched with 57 dyscalculic individuals on age, gender, country of residence, and education. 2 missing values were found across self-report measures. However, Little’s (1988) test for Missing Completely at Random (MCAR) (*χ*^2^ (22)=31.93, *p*=.079), showed that data was missing at random and no imputation of missing values was required. Population characteristics, descriptive statistics and independent *t* test results comparing the neurotypical and dyscalculic individuals on all measures are presented in Table S11 and S12.

**Table S11**

*Description of the study population in relation to the demographic variables used for matching (age, gender, level of education and country of residence)*

|  | **NT (n)** | **Dyscalculia (n)** | **Total** | **Statistics** |
| --- | --- | --- | --- | --- |
| **Age** |  |  |  | *X*^2^ (2) =0.04, *p* = .983 |
| 18-21 | 44 | 44 | 88 |  |
| 22-25 | 11 | 12 | 23 |  |
| 26+ | 1 | 1 | 2 |  |
| **Gender** |  |  |  | *X*^2^ (1) =0.03, *p* = .867 |
| Male | 13 | 14 | 27 |  |
| Female | 43 | 43 | 86 |  |
| **Education** |  |  |  | *X*^2^ (3) = 0.33, *p* = .955 |
| 1^st^ year | 29 | 29 | 58 |  |
| 2^st^ year | 13 | 13 | 26 |  |
| 3^rd^ yea | 13 | 13 | 26 |  |
| 4^th^ year | 1 | 2 | 3 |  |
| **Country of Residence** |  |  |  | *X*^2^ (10) =0 .99, *p* > .999 |
| Australia | 0 | 1 | 1 |  |
| Belgium | 3 | 3 | 6 |  |
| Canada | 1 | 1 | 2 |  |
| Egypt | 30 | 30 | 60 |  |
| England | 10 | 10 | 20 |  |
| Germany | 3 | 3 | 6 |  |
| Indonesia | 1 | 1 | 2 |  |
| Italy | 1 | 1 | 2 |  |
| Netherlands | 5 | 5 | 10 |  |
| Northern Ireland | 1 | 1 | 2 |  |
| Ukraine | 1 | 1 | 2 |  |

**Note:** *X*^2^ *= chi-square; all Ns* = 113

**Table S12.**

*Independent t tests comparing the neurotypical (n = 56) and dyscalculic (n = 57) individuals*

|  | **Neurotypical** | | **Dyscalculia** | |  |  |
| --- | --- | --- | --- | --- | --- | --- |
| **Measures** | *M (SD)* | *Range* | *M (SD)* | *Range* | *p* | *Cohen’s d* |
| Statistics Anxiety | 66.14 (21.09) | 24-114 | 74.21 (20.96) | 23-107 | .045* | 0.38 |
| Mathematics Anxiety | 68.61 (19.73) | 24-103 | 83.34 (18.55) | 39-114 | <.001** | 0.77 |
| Cognitive and Somatic Anxiety | 47.96 (15.46) | 22-84 | 51.18 (14.66) | 24-80 | .260 | 0.21 |
| Test Anxiety | 60.93 (16.71) | 28-98 | 64.14 (16.18) | 34-99 | .302 | 0.20 |
| Fear of Negative Evaluation | 22.39 (8.90) | 8-40 | 26.11 (8.53) | 9-40 | .026* | 0.43 |
| Social Anxiety | 58.50 (15.33) | 24-87 | 63.72 (13.68) | 35-88 | .059 | 0.36 |
| Creativity Anxiety | 45.43 (12.22) | 21-78 | 47.30 (10.01) | 25-66 | .376 | 0.17 |
| Intolerance of Uncertainty | 35.20(10.18) | 14-59 | 37.98(7.93) | 23-56 | .108 | 0.31 |
| General Self-Efficacy | 28.59 (5.30) | 18-40 | 28.30 (4.66) | 14-37 | .757 | 0.06 |
| Attitude Towards Mathematics | 24.30 (4.06) | 15-35 | 22.46 (4.41) | 11-33 | .022* | 0.44 |
| CRT (Deliberative response) | 0.66 (0.77) | 0-3 | 0.30 (0.65) | 0-3 | .008** | 0.51 |
| CRT (Intuitive response) | 2.14 (0.92) | 0-3 | 2.26 (0.88) | 0-3 | .479 | 0.13 |

**Note:** **p* < .05, ***p* < .01; CRT = Cognitive Reflection Test

To further investigate the predictors of group membership, we conducted a hierarchical regression analysis. In the regression model, we used neurotypical/dyscalculic as the outcome variable (0 = neurotypical; 1 = dyscalculic), with all other measures as predictors, and added age and gender (-1 = male; 1 = female) as covariates. Given that some variables (especially the anxiety measures) were highly correlated, we also computed multicollinearity statistics. The analysis showed that the VIF value for each predictor was acceptable (VIF < 3). In addition, the model met the conditions of independence of residuals. As a result, it was possible to estimate regression weights with confidence.

A significant effect was observed in the model (*F* (13, 97) = 2.07, *p* = .023, *η*^2^ = .217). The analysis showed that after controlling for the effect of age and gender, higher mathematics anxiety increased the probability of being dyscalculic. The remaining variables were non-significant predictors (see Table S13).

**Table S13.**

*Hierarchical Regression predicting group membership by demographics, anxiety measures, and CRT*

| **Dependent variable: Dyscalculia status (0, 1)** | | | | |
| --- | --- | --- | --- | --- |
|  | ***b*** | ***beta*** | ***t*** | ***p Adj R^2^*** |
| **Step 1 .018** | | | | |
| Age | 0.03 | 0.02 | 0.25 | .802 |
| Gender | 0.01 | -0.01 | -0.07 | .947 |
| **Step 2 .112** | | | | |
| Age | 0.06 | 0.05 | 0.54 | .590 |
| Gender | -0.02 | -0.03 | -0.31 | .760 |
| Statistics Anxiety | <0.01 | -0.13 | -0.92 | .360 |
| Mathematics Anxiety | 0.01 | 0.39 | 2.71 | .008** |
| Cognitive and Somatic Anxiety | <0.01 | -0.09 | -0.64 | .522 |
| Test Anxiety | -0.01 | -0.24 | -1.57 | .119 |
| Fear of Negative Evaluation | 0.01 | 0.15 | 1.09 | .276 |
| Social Anxiety | <0.01 | 0.09 | 0.62 | .536 |
| Creativity Anxiety | <0.01 | -0.07 | -0.67 | .507 |
| Intolerance of Uncertainty | 0.01 | 0.13 | 0.96 | .339 |
| General Self-Efficacy | <0.01 | -0.01 | -0.13 | .898 |
| Attitude Towards Mathematics | -0.02 | -0.13 | -1.28 | .203 |
| Cognitive Reflection Test | -0.13 | -0.20 | -1.80 | .075 |

**Note.** *b*= unstandardized regression weights, *beta*=standardized regression weights; CRT = Cognitive Reflection Test

## Analyses relating to Autism

16 neurotypical participants were matched with 16 autistic individuals on age, gender, country of residence, and education. Population characteristics, descriptive statistics and independent *t* test results comparing the neurotypical and autistic individuals on all measures are presented in Table S14 and S15.

**Table S14**

*Description of study population in relation to demographic variables used for matching (age, gender, level of education and country of residence)*

|  | **N (Neurotypical)** | **N (Autism)** | | **Total- N** | | **Statistics** | |  |
| --- | --- | --- | --- | --- | --- | --- | --- | --- |
| **Age** |  | |  | |  | | *X*^2^ (2) <0.001, *p* = 1.00 | |
| 18-21 | 11 | | 11 | | 22 | |  | |
| 22-25 | 2 | | 2 | | 4 | |  | |
| 26+ | 3 | | 3 | | 6 | |  | |
| **Gender** |  | |  | |  | | *X*^2^ (1) <0.001, *p* = 1.00 | |
| Male | 2 | | 2 | | 4 | |  | |
| Female | 14 | | 14 | | 28 | |  | |
| **Education** |  | |  | |  | | *X*^2^ (2) <0.001, *p* = 1.00 | |
| 1^st^ year | 8 | | 8 | | 16 | |  | |
| 2^st^ year | 6 | | 6 | | 12 | |  | |
| 3^rd^ year | 2 | | 2 | | 4 | |  | |
| **Country of Residence** |  | |  | |  | | *X*^2^ (3) <0.001, *p* = 1.00 | |
| Australia | 2 | | 2 | | 4 | |  | |
| England | 12 | | 12 | | 24 | |  | |
| Estonia | 1 | | 1 | | 2 | |  | |
| Netherlands | 1 | | 1 | | 2 | |  | |

**Note.** *X*^2^ *= chi-square; all Ns* = 32

**Table S15.**

*Independent t tests comparing the Neurotypical (N=16) and Autistic (N=16) individuals*

|  | **Neurotypical** | | **Autism** | | |  |  |
| --- | --- | --- | --- | --- | --- | --- | --- |
| **Measures** | *M (SD)* | *Range* | | *M (SD)* | *Range* | *p* | *Cohen’s d effect sizes* |
| Statistics Anxiety | 75.44 (24.55) | 34-109 | | 81.13 (14.88) | 49-101 | .434 | 0.28 |
| Mathematics Anxiety | 73.06 (25.34) | 38-115 | | 86.50 (17.88) | 47-116 | .093 | 0.61 |
| Test Anxiety | 65.81 (22.33) | 23-65 | | 70.50 (16.73) | 28-82 | .295 | 0.38 |
| Cognitive and Somatic Anxiety | 46.50 (14.77) | 36-88 | | 52.44 (16.67) | 44-93 | ..507 | 0.24 |
| Fear of Negative Evaluation | 25.63 (8.99) | 12-40 | | 30.75 (8.00) | 9-40 | .099 | 0.60 |
| Social Anxiety | 62.63 (15.79) | 32-86 | | 70.38 (12.95) | 49-92 | .140 | 0.54 |
| Creativity Anxiety | 42.38 (14.32) | 16-72 | | 46.75 (9.56) | 28-71 | .319 | 0.36 |
| Intolerance of Uncertainty | 35.31 (11.48) | 17-44 | | 46.25 (8.43) | 29-60 | .005** | 1.09 |
| General Self-Efficacy | 28.31 (4.60) | 19-36 | | 22.75 (6.62) | 11-37 | .214 | 0.45 |
| Attitude Towards Mathematics | 23.00 (5.38) | 18-32 | | 24.38 (4.01) | 16-30 | .420 | 0.29 |
| CRT (Deliberative response) | 1.19 (0.91) | 0-2 | | 0.81 (0.98) | 0-3 | .271 | 0.40 |
| CRT (Intuitive response) | 1.63 (0.89) | 1-3 | | 2.00 (1.03) | 0-3 | .279 | 0.39 |

**Note** ***p* < .01; CRT = Cognitive Reflection Test

## Analyses relating to dyspraxia

Fourteen neurotypical participants were matched with 14 dyspraxic individuals based on age, gender, country of residence, and education. Population characteristics, descriptive statistics and independent *t* test results comparing the neurotypical and dyspraxic individuals on all measures are presented in Table S16 and S17.

**Table S16**

*Description of study population in relation to demographic variables used for matching (age, gender, level of education and country of residence)*

|  | **N (Neurotypical)** | **N (Dyspraxia)** | **Total N** | **Statistics** |
| --- | --- | --- | --- | --- |
| **Age** |  |  |  | *X*^2^ (2) < 0.001, *p* = 1.00 |
| 18-21 | 11 | 11 | 22 |  |
| 22-25 | 2 | 2 | 4 |  |
| 26+ | 1 | 1 | 2 |  |
| **Gender** |  |  |  | *X*^2^ (1) <0.001, *p* = 1.00 |
| Male | 8 | 8 | 16 |  |
| Female | 6 | 6 | 12 |  |
| **Education** |  |  |  | *X*^2^ (2) <0.001, *p* = 1.00 |
| 1^st^ year | 7 | 7 | 14 |  |
| 2^st^ year | 5 | 5 | 10 |  |
| 3^rd^ year | 2 | 2 | 4 |  |
| **Country of Residence** |  |  |  | *X*^2^ (3) <0.001, *p* = 1.00 |
| Egypt | 2 | 2 | 4 |  |
| England | 10 | 10 | 20 |  |
| Northern island | 1 | 1 | 2 |  |
| Scotland | 1 | 1 | 2 |  |

**Note.** *X*^2^ *= chi-square; all Ns* = 28

**Table S17**

*Independent t tests comparing the Neurotypical (N=14) and Dyspraxic (N=14) individuals*

|  | **Neurotypical** | | | **Dyspraxia** | |  |  |
| --- | --- | --- | --- | --- | --- | --- | --- |
| **Measures** | *M (SD)* | *Range* | *M (SD)* | | *Range* | *p* | *Cohen’s d effect sizes* |
| Statistics Anxiety | 65.79 (22.19) | 50-97 | 74.36 (19.59) | | 39-103 | .289 | 0.41 |
| Mathematics Anxiety | 71.29 (23.16) | 57-103 | 78.14(24.00) | | 38-120 | .449 | 0.29 |
| Test Anxiety | 50.29 (16.83) | 30-93 | 73.50(17.15) | | 45-100 | <.001** | 1.37 |
| Cognitive and Somatic Anxiety | 45.21 (9.97) | 29-80 | 53.00(11.56) | | 36-80 | .067 | 0.72 |
| Fear of Negative Evaluation | 21.64 (10.61) | 11-40 | 28.93(9.89) | | 11-40 | .071 | 0.71 |
| Social Anxiety | 60.14 (19.91) | 35-92 | 65.00(13.38) | | 46-96 | .456 | 0.29 |
| Creativity Anxiety | 40.86 (14.68) | 17-64 | 42.64(11.32) | | 26-62 | .721 | 0.14 |
| Intolerance of Uncertainty | 33.36 (8.28) | 24-57 | 40.86 (9.63) | | 25-60 | .036* | 0.84 |
| General Self-Efficacy | 26.43 (5.72) | 21-36 | 25.86 (5.56) | | 17-33 | .791 | 0.10 |
| Attitude Towards Mathematics | 23.64 (5.33) | 19-34 | 24.71 (4.18) | | 16-31 | .559 | 0.22 |
| CRT (Deliberative response) | 1.29 (1.33) | 0-3 | 1.50 (1.16) | | 0-3 | .653 | 0.17 |
| CRT (Intuitive response) | 1.36 (1.34) | 0-3 | 1.07 (1.14) | | 0-3 | .548 | 0.23 |

**Note.** **p* < .05, ***p* < .01; CRT = Cognitive Reflection Test
